# Integrative Identification of Crucial Genes Associated With Plant Hormone-Mediated Bud Dormancy in *Prunus mume*

**DOI:** 10.3389/fgene.2021.698598

**Published:** 2021-07-06

**Authors:** Ping Li, Tangchun Zheng, Zhiyong Zhang, Weichao Liu, Like Qiu, Jia Wang, Tangren Cheng, Qixiang Zhang

**Affiliations:** ^1^Beijing Key Laboratory of Ornamental Plants Germplasm Innovation & Molecular Breeding; National Engineering Research Center for Floriculture; Beijing Laboratory of Urban and Rural Ecological Environment; Engineering Research Center of Landscape Environment of Ministry of Education; Key Laboratory of Genetics and Breeding in Forest Trees and Ornamental Plants of Ministry of Education, Beijing Forestry University, Beijing, China; ^2^School of Landscape Architecture, Beijing Forestry University, Beijing, China; ^3^Department of Hematology, The Second Affiliated Hospital, Guangzhou Medical University, Guangzhou, China

**Keywords:** *Prunus mume*, dormancy, co-expression network, ABA, expression and function

## Abstract

*Prunus mume* is an important ornamental woody plant with winter-flowering property, which is closely related to bud dormancy. Despite recent scientific headway in deciphering the mechanism of bud dormancy in *P. mume*, the overall picture of gene co-expression regulating *P. mume* bud dormancy is still unclear. Here a total of 23 modules were screened by weighted gene co-expression network analysis (WGCNA), of which 12 modules were significantly associated with heteroauxin, abscisic acid (ABA), and gibberellin (GA), including GA1, GA3, and GA4. The yellow module, which was positively correlated with the content of ABA and negatively correlated with the content of GA, was composed of 1,426 genes, among which 156 transcription factors (TFs) were annotated with transcriptional regulation function. An enrichment analysis revealed that these genes are related to the dormancy process and plant hormone signal transduction. Interestingly, the expression trends of *PmABF2* and *PmABF4* genes, the core members of ABA signal transduction, were positively correlated with *P. mume* bud dormancy. Additionally, the *PmSVP* gene had attracted lots of attention because of its co-expression, function enrichment, and expression level. *PmABF2*, *PmABF4*, and *PmSVP* were the genes with a high degree of expression in the co-expression network, which was upregulated by ABA treatment. Our results provide insights into the underlying molecular mechanism of plant hormone-regulated dormancy and screen the hub genes involved in bud dormancy in *P. mume*.

## Introduction

Dormancy is a strategy in which higher plants survive under adverse conditions by suspending growth and development (Holdsworth et al., 2008). When the seasons change—for instance, from summer to autumn—plants will face environmental changes such as lower temperature, shorter days, and lower ratios of red (R) and far-red (FR) light (de Wit et al., 2013; Kegge et al., 2015). By integrating various environmental stimuli and *in vivo* signal responses to balance growth and dormancy, plants show seasonal adaptability for long-term evolution. The two main signals that plants depend on to respond to seasonal changes were photoperiod and temperature, which play a key role in the growth–dormancy cycle of trees (Maurya and Bhalerao, 2017). Plenty of studies have reported that photoperiod and temperature play crucial but opposite roles in the induction and release of dormancy, but the thresholds and combined effects of these environmental factors remain to be determined. Studies on peach showed that a short photoperiod can induce bud dormancy under non-low-temperature conditions, whereas low temperature promotes bud dormancy under a long-photoperiod condition (Li S. et al., 2018). If the photoperiod is shorter than the critical threshold for plant growth (short day), the growth will stop and the terminal bud will eventually form, surrounding the shoot tip meristem, while short day will cause the bud to transit to dormancy (Kainer et al., 1991; Barrero et al., 2012).

In previous studies, the core components of ABA biosynthesis and signal transduction have been identified by molecular genetics and biochemical and pharmacological methods (Chen et al., 2020). ABA-mediated dormancy is usually associated with different degrees and types of seed dormancy, while bud dormancy is scarce (Shu et al., 2016; Chen et al., 2020; Zhou et al., 2020). Meanwhile, ABA-mediated bud dormancy has been explored more in woody plants than in herbaceous plants, such as poplar, grape, and peach (Holdsworth et al., 2008; Li and Dami, 2015; Li S. et al., 2018; Tylewicz et al., 2018). Light signals are closely related to plant hormones, some of which are involved in the induction or release of winter dormancy, thus regulating the adaptability of plants to the environment. Short day induced the expression of abscisic acid (ABA) receptors in hybrid poplar and increased the level of ABA in bud, suggesting that ABA responded to short-photoperiod-mediated plasmodesmata closure and further induced bud dormancy (Lau and Deng, 2010; Singh et al., 2019). At the same time, short day induced plasmodesmata D closure by enhancing the ABA response, prevented the transmission of growth signals, and maintained bud dormancy (Singh et al., 2019). The photoperiodic control of ABA-mediated dormancy reveals that *SHORT VEGETATIVE PHASE-LIKE* (*SVL*) induces *CALLOSE SYNTHASE* expression to further induce plasmodesmatal closure and negatively regulates the gibberellin (GA) pathway to promote dormancy (Singh et al., 2018, 2019). The ecotopic expression of *SVP* affects the dormancy duration of cold-tolerant kiwifruit, has a minimal effect on the dormancy duration of cold-sensitive kiwifruit, and suggests a complementary role with ABA (Wu et al., 2017). In grape, *VvSnRK1* regulates the dormancy induction and regulation of metabolic changes through both ABA synthesis and activities (Parada et al., 2016). During leafy spurge dormancy, the content of ABA and PA decreased, while the level of heteroauxin (IAA) remained unchanged (Chao et al., 2017). Ethylene and ABA regulate the biosynthesis of ascorbic acid by regulating EIN3 and ABI4 (Bossi et al., 2009; An et al., 2010). The opposing roles of ABA and GA in dormancy result in a balanced control mechanism, and other plant hormone signal transduction and synthesis also contribute to this balance (Liu and Hou, 2018; Chen et al., 2020).

The gene expression profiles are effectively and comprehensively described using high-throughput transcriptome sequencing technologies. The RNA-seq technology has been widely applied on a range of systems in many studies of plants, such as stress, growth, and development (Moenga et al., 2020; Swift et al., 2020; Sun et al., 2021). However, previous transcriptome studies have usually focused on the identification and screening of differentially expressed genes, while the degree of interconnection between related genes has not yet been considered. Since genes with similar expression patterns may have the same function, the identification of these genes can provide more information about their corresponding possible molecular regulatory mechanisms (Yuan et al., 2018; Farhadian et al., 2021). WGCNA can be used to construct the co-expression networks by gene expression profiles (Langfelder and Horvath, 2008; Presson et al., 2008). WGCNA has been reported to be used to investigate the co-expression network and hub genes involved mainly in plants under abiotic stress, such as salt, drought and cold stress (Cheng et al., 2020; Moenga et al., 2020; Panahi and Hejazi, 2021). More recently, the effectiveness of this approach for deciphering and disentangling the complex process has been proven, thereby highlighting the power of the co-expression networks to provide deep insights into these complex processes (Panahi et al., 2020; Farhadian et al., 2021; Panahi and Hejazi, 2021).

*Prunus mume* is a very important ornamental woody plant, providing quality material for ecotope-based landscape design, and its contribution to the public environment and urban landscaping continues to increase daily. *P. mume* is an excellent material for studying plant bud dormancy because of its winter-flowering property. In our previous study, *PmDAM* and *PmCBF* genes are involved in the molecular regulation of *P. mume* bud dormancy, while PmCBF1 transcription factor can form homodimer and heterodimer with PmCBF6 and heterodimer with three members of the PmDAM family (PmDAM1/2/6). PmCBFs are involved in the regulation of *PmDAM6* gene expression by binding to *cis-*acting elements (Xu et al., 2014; Zhao et al.,2018a,b, c). Based on the transcriptome profile that reveals the key roles of hormones and sugars, a hypothetical model was proposed to understand the molecular mechanisms of dormancy in *P. mume* (Zhang et al., 2018). Despite recent scientific headway in deciphering the mechanism of bud dormancy in *P. mume*, the overall picture of gene co-expression regulating *P. mume* bud dormancy is still unclear. In this study, we established the co-expression network in *P. mume* bud dormancy and aided in clarifying the mechanisms regulating dormancy in *P. mume*. Moreover, the genes identified here can serve as valuable genetic resources or selection targets for further molecular breeding of *P. mume*.

## Materials and Methods

### Data Collection

To further investigate the correlation between gene expression and plant hormones, we collected transcriptome data and plant hormone content during *P. mume* bud dormancy. The dormancy process was divided into four stages according to the percentage bud break. All samples from *P. mume* var. “Zaohua Lve,” including the four stages of dormancy, were three biological replicates from independent individuals. The RNA-seq datasets of 12 samples were obtained using the Illumina HiSeq^TM^4000 platform. Meanwhile, we collected the plant hormone content at these four stages from *P. mume* var. “Zaohua Lve” (Zhang et al., 2018). The plant hormonal quantification, including NAA, ABA, GA1, GA3 and GA4, was measured using HPLC–ESI–MS/MS with a standard measure. *P. mume* genome was obtained from the *P. mume* genome project (Zhang et al., 2012) for gene annotation.

### Co-expression Network Construction

First, the samples and genes were filtered according to the gene expression profiles. Here we removed genes and samples with the absent rate greater than or equal to 10%. Co-expression network and module identification were analyzed for filtered dataset using the WGCNA package (Langfelder and Horvath, 2008). In order to maintain the gene connectivity and greater weight to strongest correlations, the soft threshold of the correlation matrix (β) was selected. The β value was calculated using the “pickSoftThreshold” function in the WGCNA package. The “blockwiseModules” function is one-step network construction and module detection. The adjacency matrix was converted to topological overlap matrix, and the topological overlap measure was calculated. The topological overlap matrix used dissimilarity between genes to cluster genes, and then it used dynamic tree cutting algorithm to shear the trees into different modules. Then, the co-expression network was constructed, and 400 genes were randomly selected to draw topologically overlapping heat maps.

### Correlation Analysis of Gene Expression Level and Plant Hormone Content

The content of plant hormonal quantification was used as trait data. The correlation coefficients between each module eigengene and phenotype were calculated using the “cor” and “corPvalueStudent” functions. Module–trait associations were estimated based on the correlation between the module eigengene and the phenotype, and the associated heat map was drawn using the “labeledHeatmap” function. Modules with a correlation greater than or equal to 0.9 and a minimum *P*-value were selected in the module–trait associations.

### Enrichment Analysis of Genes Within Network Modules

Genome-wide Gene Ontology (GO) and Kyoto Encyclopedia of Genes and Genomes (KEGG) annotation files were constructed using *P. mume* genomes, respectively. Based on the GO database (Gene Ontology, 2021), the genes within the module were annotated through the GO online tool^1^, including molecular function, cellular component, and biological process. Based on the KEGG database (Kanehisa et al., 2021), the genes within the module were enriched through the KEGG online tool^2^. R language was used for statistics and visualization of enrichment results.

### Expression Trend Analysis and TF Enrichment

To investigate patterned differences in expression profiles among modules, genes within the module were identified for all possible expression trends at four stages. The enriched sequences were extracted using TBtool software (Chen et al., 2018). Meanwhile, the interaction network of enriched TFs was constructed according to the orthologs of *Arabidopsis* using STRING (Szklarczyk et al., 2017). Cytoscape software was used for visualization of the network (Smoot et al., 2011). The symbol and description of genes were annotated through UniProtKB and NCBI databases, respectively (Johnson et al., 2008; UniProt, 2019).

### ABA Treatment and Relative Gene Expression Analysis

*Prunus mume* var. “Zaohua Lve” from grafted plants with the same genotype was cultivated in the greenhouse. For ABA treatment, the plants were sprayed with 50 μM ABA, and water was used as the control. The samples were collected 24 h after the treatment, frozen in liquid nitrogen, and stored at −80°C before use.

Total RNA extraction and cDNA synthesis were performed according to previous methods (Zhuo et al., 2018). We designed specific primers for qRT-PCR using INTEGRATED DNA TECHNOLOIES tool^3^. The detailed information of the primers are shown in Supplementary Table 1. The gene expression level was measured using the CFX96 Real-Time PCR Detection System with TB Green^TM^ Premix Ex *Taq*^TM^ II (TaKaRa, Bejing, China). The relative gene expression level was calculated using the 2^–ΔΔ^*^Ct^* method (Livak and Schmittgen, 2001).

## Results

### Construction of Co-expression Network and Correlation Between Modules and Hormone Content

**FIGURE 1:**
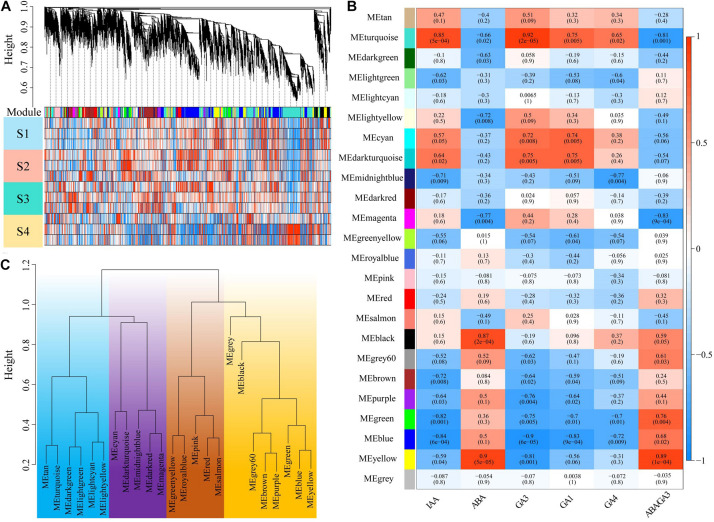
Identification of key modules correlated with plant hormone content in the RNA-seq dataset through weighted gene co-expression network analysis. **(A)** The gene dendrogram and the corresponding module colors. The clustering was based on the RNA-seq data in four stages of dormancy. **(B)** A heat map showing the relationship between the modules and plant hormones. The corresponding correlation and *p*-value were presented in the first line and second line of each cell, respectively. **(C)** Clustering of module eigengenes. Clustering was distinguished by different colors.

To assess how the change of plant hormone content contributes to *P. mume* bud dormancy, WGCNA was applied to investigate gene sets that were related to plant hormone content using the RNA-seq during the dormancy period. After filtering, a total of 14,983 genes were selected based on their expression levels throughout the 16 samples. A scale-free network was constructed with the soft-threshold β = 12 based on the cutoff of *R*^2^ = 0.9 (Figure 1A and Supplementary Figure 1). We randomly selected 400 genes to construct an interactive relationship network, which further verified the reliability of the module (Supplementary Figure 2). We identified 23 distinct co-expression modules using the dynamic tree cutting algorithm, each consisting of 35 to 3,825 correlated genes, of which 12 modules were significantly associated with IAA, ABA, GA1, GA3, and GA4. Most of the gene co-expression modules showed an inverse relationship between ABA and GA, but there were also some hormone specific co-expression gene modules—for example, the yellow module, including 1,426 genes, was most positively related to ABA (*r* = 0.9, *P* = 5e-5), whereas it was negatively correlated with the GA3 (*r* = −0.8, *P* = 0.001). Nevertheless, the black module was only significantly correlated with ABA (*r* = 0.87, *P* = 2e-4) (Figure 1B). The GA significantly correlated modules had a correlation relationship similar to that of the co-expression modules, in which GA3 had a stronger correlation among these modules than GA1 and GA4, suggesting that the biosynthetic genes and expression regulation genes of GA3 might play a more important role in *P. mume* bud dormancy. Furthermore, the eigengene dendrogram showed that these modules, including the gray module without the correlated genes, could be divided into four major branches (Figure 1C), indicating that co-expression patterns with varying functions were present in the networks.

### Functional Enrichment of the Gene Module Analysis

**FIGURE 2:**
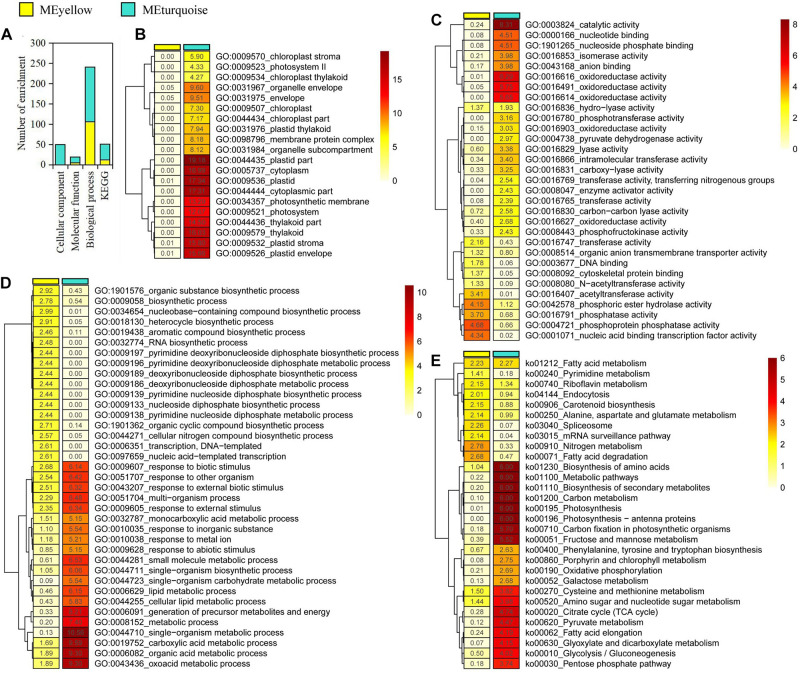
Gene enrichment analysis in yellow and turquoise modules. **(A)** Statistics of significantly enriched genes. **(B–E)** Heat maps of -log10 enrichment *p*-values for the 20 most-enriched Gene Ontology terms and Kyoto Encyclopedia of Genes and Genomes pathway in the shared genes.

We focused on the yellow and turquoise modules that were significantly associated with ABA and GA for functional enrichment analysis. In the yellow and turquoise modules, 111 and 199 GO terms were identified in three GO groups (cellular component, molecular function, and biological process), respectively (Figure 2A and Supplementary Tables 2–7). In three types of GO terms, more than half of the gene enrichment belonged to the biological process (Figure 2A), while genes from the yellow module did not have significantly enriched GO terms in the cellular component (Figure 2B). The molecular function of the selected genes in the yellow modules revealed an enrichment of the cell protein phosphorylation state, including phosphoprotein phosphatase activity, phosphoric ester hydrolase activity, and phosphatase activity, suggesting that these genes were involved in regulating cell activity. Meanwhile, the molecular function of the turquoise module was mostly enriched in catalytic activity, oxidoreductase activity, and nucleoside phosphate binding (Figure 2C). In biological process, the yellow and turquoise module presented obvious differences in enriched GO terms but also contained the same enriched GO terms, such as response to biotic stimulus (Figure 2D). The biological process of the yellow module was mostly enriched in nucleobase-containing compound biosynthetic process, organic substance biosynthetic process, and heterocycle biosynthetic process. Additionally, GO terms associated with plant dormancy were significantly enriched, including dormancy process and seed dormancy process (Supplementary Tables 6, 7).

Furthermore, the pathway enrichment analysis of genes from the yellow and turquoise modules, respectively, was conducted using KEGG database. As a result, we discovered a number of very important biosynthetic and metabolic pathways, such as carbon metabolism and fatty acid biosynthesis (Figure 2E). Notably, plant hormone signal transduction was enriched in the modules, which contained 43 genes in the yellow module and 107 genes in the turquoise module, respectively (Supplementary Tables 8, 9). In plant hormone signal transduction, the enriched genes in the yellow module included abscisic acid receptor PYR/PYL family (*PYL*), protein phosphatase 2C (*PP2C*), and ABA-responsive element binding factor (*ABF*) genes involved in ABA signal transduction pathway. Meanwhile, the enriched genes in the turquoise module included gibberellin receptor GID1 (*GID1*), F-box protein GID2 (*GID2*), and DELLA protein (*DELLA*) genes, which are involved in GA signal transduction pathway. In addition, some genes that involved cytokinin, gibberellin, jasmonic acid, brassinolide, and auxin signal transduction pathways were identified in these enriched genes, suggesting that there were elaborate signaling networks and complex cross-talking among different hormone signal transduction pathways. Based on GO and KEGG enrichment results, we speculated that ABA- and GA3-related genes played important regulatory roles but presented different regulatory mechanisms in *P. mume* bud dormancy.

### Expression Trend Analysis

**FIGURE 3:**
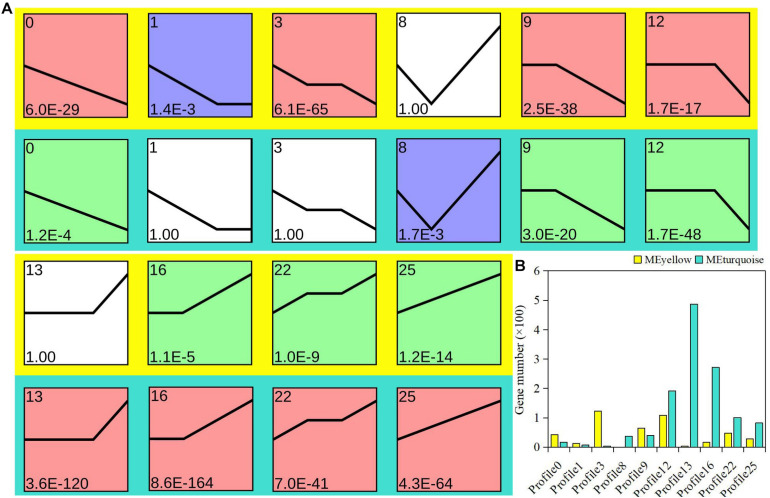
Analysis of gene expression trend in yellow and turquoise modules. **(A)** Each plot shows the median expression value of all genes with a similar expression profile during *Prunus mume* bud dormancy. **(B)** Statistics of gene quantity in profiles.

To explore the possible expression pattern of the module-related genes in bud dormancy, expression trend analysis was performed to further divide the module-related genes into 26 profiles. Ten profiles (colored block) were divided into three clusters with clear and distinct expression profiles (Figure 3A). On the whole, the gene expression pattern between the yellow and turquoise modules showed an opposite trend. In the yellow module, 75.8% of the genes showed a downregulated trend, attributed to the red cluster, including profiles 0, 3, 9, and 12. However, 76.1% of the genes were upregulated in the turquoise module (Figure 3B). The expression trends of 136 genes, belonging to profiles 1 and 3, were specifically found in the yellow module. Similarly, there were specific trend profiles in the turquoise module, among which profile 13 contained most genes. Based on gene expression trends, we speculated that these downregulated genes from the yellow module might be actively involved in the regulation of *P. mume* bud dormancy. Therefore, as was also evident in our analysis of gene enrichment, the enrichment of functional differences in these two important modules was probably associated with expression pattern and represented plant-hormone-specific regulatory mechanisms in dormancy.

### A Network of Expression and Transcriptional Regulation in Modules

**FIGURE 4:**
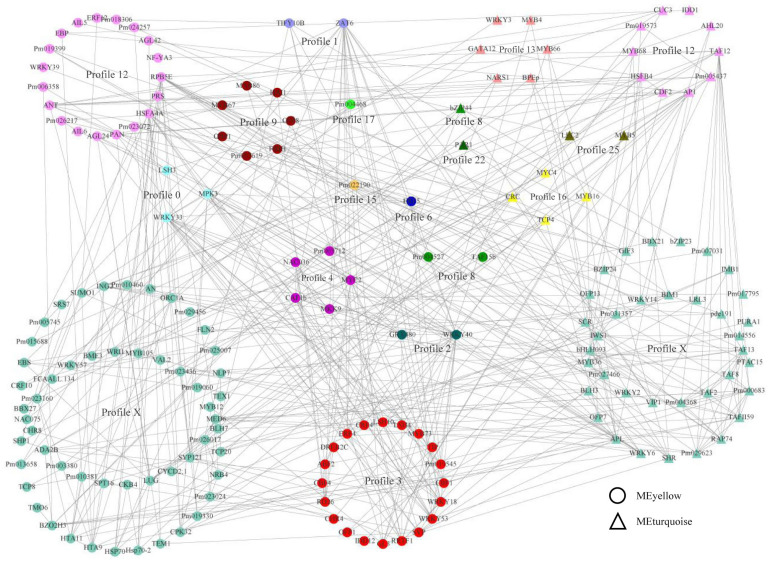
A network of expression and transcriptional regulation. The transcription factors in the turquoise module were noted with triangles, and those in the yellow module were noted with circles. The colors indicated the trends in which the genes were expressed.

To estimate the crucial genes by weighted gene co-expression network and to better rank expression and regulatory interactions, a total 5,251 genes from the yellow and turquoise modules were annotated according to the orthologous genes of *Arabidopsis thaliana*. TFs played a more extensive role in gene expression, which was mainly controlled *via* the interactions with promoters and chromatin-modifying machinery (Liu and Stewart, 2016; Moenga et al., 2020). In this study, we obtained 156 and 76 TFs in the yellow and turquoise modules, respectively, which were co-expressed with all genes in the modules. The TF interaction network was predicted by the string database and hid disconnected nodes. Therefore, a network of expression and transcriptional regulation about hub TFs was constructed, combining co-expression relationships and interaction networks (Figure 4). The network showed that TFs from different modules with different expression modes also had close regulatory relationships. Profile X, with no significant enrichment in the gene expression pattern, contained the most TFs and interacted with other TFs, suggesting that these TFs might not be involved in bud dormancy regulation but could interact with TFs involved in bud dormancy regulation and participate in other life activities. We found that most transcription factors were associated with abiotic stress, growth and development, and hormonal responses, such as *WRKY*, *bZIP*, *AP2/ERE*, and *MYB* genes. Interestingly, the TFs of different modules in the same profile (profile 12) had different molecular functions, with the TF function in the yellow module preferring dormancy and abiotic stress, while the TF function in the blue module preferred transition to germination, flowering, and shoot development. In profile 3, the functional annotation of each TF was most closely related, mainly including cold stress response (*CBF*, *CRF*, and *WRKY*), plant hormone (*ABF*, *ERF*, and *MYB*), and light response (*SVP* and *LSH*). These results indicated that plant-hormone-related TFs were involved in the regulation of plant dormancy and that they play an important role in the network of expression and transcriptional regulation.

### Identification of Hub Genes and Expression Analysis

**FIGURE 5:**
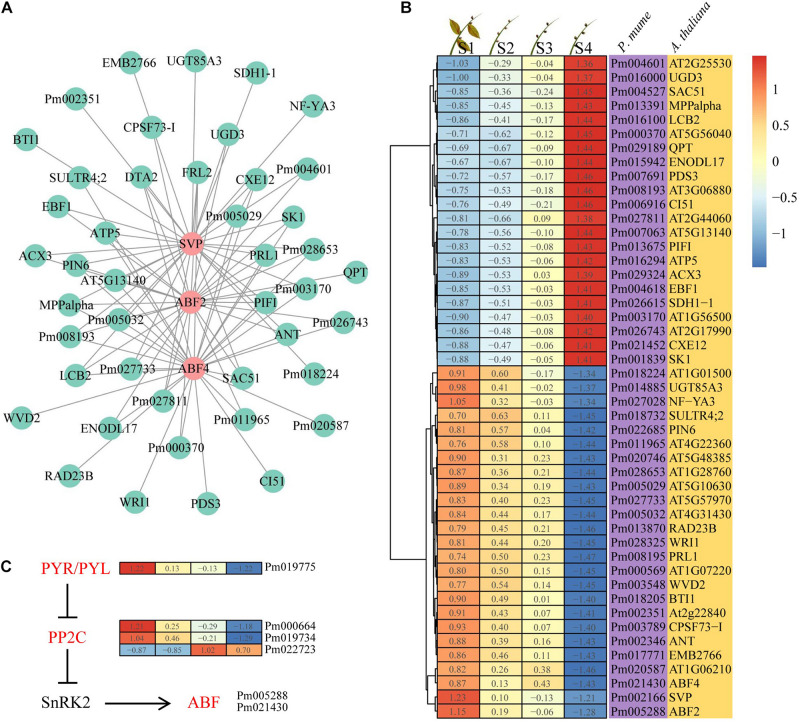
Interaction network and expression of the hub genes. **(A)** Interaction of gene co-expression network of the hub genes. **(B)** Heat map showing the genes that were expressed at four different dormancy stages of *Prunus mume*. **(C)** Abscisic acid (ABA) signal transduction pathway. The ABA-related genes in yellow module were highlighted in red.

Considering the expression trend and intramodular connectivity for genes in bud dormancy, three genes (*PmABF2*, *PmABF4*, and *PmSVP*) closely related to ABA were selected; they belong to profile 3 in the yellow module. *PmABF2*, *PmABF4*, and *PmSVP*, as hub genes, were co-expressed with 1,301, 1,197, and 1,305 genes in the yellow module by the WGCNA algorithm, respectively. Here the top 30 genes co-expressed with *PmABF2*, *PmABF4*, and *PmSVP* were presented based on the intramodular connectivity through a network (Figure 5A). We found that more than half of the genes were collectively co-expressed with these three genes; however, only two genes were specific in the *ABF2* gene co-expression network. The expression patterns of 47 genes in this network were divided into two categories, among which 22 genes were upregulated and 25 genes were downregulated during bud dormancy (Figure 5B), suggesting that *PmABF2*, *PmABF4*, and *PmSVP* might negatively regulate the upregulated genes. In ABA signal transduction pathway, *PmPYL* (Pm019775) and two *PmPP2C* (Pm000664 and Pm019734) genes had similar expression patterns with *PmABFs* (Figure 5C). However, PYL inhibits the activity of PP2Cs in an ABA-independent manner but more efficiently when activated by ABA (Park et al., 2009; Hao et al., 2011). These results suggested that another upregulated *PmPP2C* gene (Pm022723) might play a role in this pathway. The two clusters were composed of transcripts that were specific dormancy stages in the expression trends, and the associated *PmABF2*, *PmABF4*, and *PmSVP* provided clues about the regulatory networks.

**FIGURE 6:**
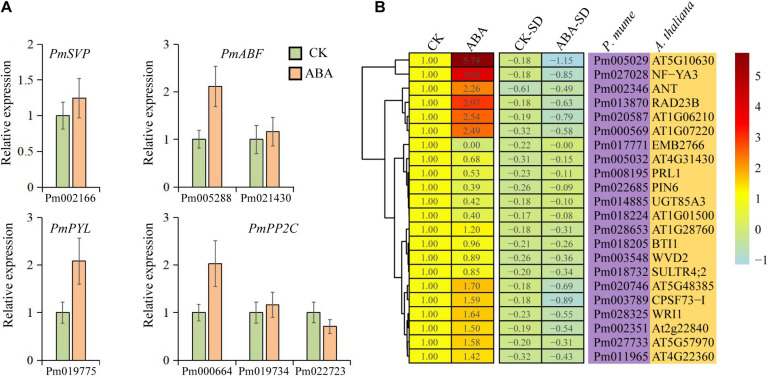
Expression patterns of candidate genes under abscisic acid (ABA) treatment. **(A)** qRT-PCR of 7 selected genes under ABA treatment. **(B)** Heatmap of 22 genes under ABA treatment. CK represents the control group, ABA represents the ABA treatment group, and CK-SD and ABA-SD represent the standard deviations of relative expression levels in the groups, respectively.

Although the intensity of expression varies among different tissue, 65.5% of these genes were upregulated with ABA treatment, compared with 34.5% downregulated transcripts (Figure 6). The expression of both *PmPYL* and *PmABF* homologs, which were involved in the first and final steps of the ABA signal transduction pathway, respectively, was induced by ABA treatment (Figure 6A). Interestingly, the ABA signal transduction pathway led to a higher expression level of *PmPYL* by ABA treatment while downregulating the *PmPP2C* gene (Pm022723), which acted as a negative regulator of seed dormancy by inhibiting ABA signaling and subsequently activating GA signaling (Kim et al., 2013). This regulatory pathway might also be applicable to bud dormancy in *P. mume*. However, other two *PmPP2C* genes were upregulated after ABA treatment, which was consistent with the level of gene expression during bud dormancy. *PmSVP*, a *P. mume* MADS-box gene, was upregulated by ABA treatment, and half of its associated genes in the co-expression network were also upregulated. The two most prominent upregulated genes, *PmIF2*γ (Pm005029, a translation elongation factor EF1A/initiation factor IF2gamma family protein) and *PmNF-YA3* (Pm027028, a nuclear transcription factor Y subunit A-3), were co-expressed with *PmABF2/4* and *PmSVP*, respectively. In downregulated genes, *EMB2766* (Pm017771) was most significantly inhibited by ABA treatment, whose function has been described as the structural constituent of nuclear pore in *Arabidopsis* (Tamura et al., 2010). At the same time, the functional annotations of some downregulated genes were related to plant growth and development, such as *PIN6* (Pm022685), an auxin efflux carrier family protein gene (Figure 6B). These gene expressions confirmed and extended the dormancy process analysis above, furthering the description of a conserved ABA treatment response.

## Discussion

Bud dormancy is a common adaptive response of perennial plants to survive in adverse environmental conditions (Tarancón et al., 2017; Liu and Sherif, 2019). Plant dormancy greatly preserves reproductive success, productivity, and survival through stalled growth and reduced metabolic activities (Tarancón et al., 2017). *P. mume* has the winter-flowering property, which also means that its bud dormancy period is relatively short. Although some cultivated species are able to tolerate temperatures as low as −19°C, it is very different from the freezing tolerance of apricot, a close relative species with a longer bud dormancy period (Zhang, 1987, 1989). Bud dormancy is likely one of the key factors in the poor freezing tolerance of *P. mume*.

Plant hormone-mediated dormancy has been extensively demonstrated, especially plant hormones ABA and GA (Liu and Sherif, 2019; Chen et al., 2020). The changes in ABA and GA content are inversely correlated during dormancy, with the ABA/GA ratio varying with the level of dormancy (Liu and Sherif, 2019). In *P. mume*, the content of ABA decreased gradually, while the content of GA increased gradually from bud dormancy through dormancy release (Wen et al., 2016; Zhang et al., 2018). The ABA signal transduction pathway has been confirmed by a large number of studies, mainly including *PYR/PYL*, *PP2C*, *SnRK2s*, and *AREB/ABF* genes (Raghavendra et al., 2010; Soon et al., 2012). The observed changes in the expression of genes involved in ABA signal transduction, including *PmPYL*, *PmPP2C*, and *PmABF*, suggest a central role for ABA during *P. mume* bud dormancy (Figure 5C). The regulation of bud dormancy by the ABA signal transduction pathway has been demonstrated in woody plants such as grape, pear, and poplar (Ruttink et al., 2007; Boneh et al., 2012; Li J. et al., 2018). ABA responses during bud dormancy may also be mediated by ABA-dependent pathways in *P. mume*. Interestingly, *PmCBF* genes, as well-known cold response conduits, are co-expressed with *PmABF* genes, belonging to profile 3 (Figure 4). CBF/DREB TFs, as well-known cold response genes, have been shown to bind directly to interact with dormancy regulator DAM TFs in an ABA-independent manner (Zhao et al.,2018b,c). These results suggest a multiple manner in the dormancy regulation of each other, as demonstrated by the TF interaction network. Based on this network, we speculate that these TFs and the regulated target genes may further regulate plant dormancy by responding to low temperature or photoperiod to cause changes in endogenous hormones. The expressions of ABA-related genes were upregulated by low temperature in grape (Boneh et al., 2012) and upregulated by short day in poplar (Singh et al., 2018; Tylewicz et al., 2018).

In addition, the molecular mechanism of ABA involvement in bud dormancy by regulating *SVP/SVL* genes has been widely reported. ABA content is increased by environmental signals, thereby inhibiting the expression of a chromodomain remodeling factor *PICKLE* (*PKL*) gene (Tylewicz et al., 2018). *PKL* negatively regulates the expression of *SVP/SVL* gene and further negatively regulates *flowering locus T* (*FT*) and GA-related gene (Singh et al., 2018, 2019). Meanwhile, SVP/SVL, acting as a MADS box, positively regulates *callose synthase 1* (*CALS1*), thereby promoting bud dormancy (Singh et al., 2019). In *P. mume*, the expression pattern of *PmSVP* gene is gradually decreasing during bud dormancy. The *PmSVP* gene is upregulated by ABA treatment, and half of its associated genes in the co-expression network are also upregulated. Taken together, the *PmSVP* gene is central to the regulation of *P. mume* bud dormancy. Short day can induce the expression of *SVL* gene, while low temperature inhibits gene expression in poplar (Singh et al., 2018, 2019).

The cell cycle also plays a key role in bud dormancy, in contrast to the dormancy mechanism of intercellular communication involving the *SVL* gene. The cells of the dormant bud stagnate in the G1 phase during the cell cycle (Cespedes et al., 1995; Gutierrez et al., 2002). ABA can maintain cells in the G1 phase by interfering with DNA replication (Swiatek et al., 2002; Vergara et al., 2017). In the yellow module, *PmCYCD2;1* (Pm002137) is enriched during *P. mume* bud dormancy, which is negatively correlated with ABA content. CYCD proteins interact with cyclin-dependent kinases, which is activated by cell division cycle protein (Riou-Khamlichi et al., 1999, 2000). In grape bud dormancy, ABA represses the expression of cell cycle genes, including two *VvCDKB*s, three *VvCYCA*s, *VvCYCB*, and *VvCYCD3.2a* (Vergara et al., 2017). According to current studies, the molecular mechanism of bud dormancy is mainly based on ABA, which has been studied from different directions.

## Conclusion

In the present study, we performed WGCNA of the association between bud dormancy and plant hormones. A total of 23 distinct co-expression modules were identified by WGCNA, each consisting of 35 to 3,825 correlated genes, of which 12 modules were significantly associated with IAA, ABA, GA1, GA3, and GA4. Most of the gene co-expression modules showed an inverse relationship between ABA and GA. The enrichment analysis revealed that these genes were related to the dormancy process, seed dormancy process, and plant hormone signal transduction. In ABA signal transduction, *PmABF2* and *PmABF4* genes, belonging to profile 3 in the yellow module, were positively correlated with *P. mume* bud dormancy. Meanwhile, *PmSVP* gene, a MADS box gene, was upregulated by ABA treatment, and half of its associated genes in the co-expression network were also upregulated. Taken together, *PmABF2*, *PmABF4*, and *PmSVP* might be the hub genes involved in the regulation of *P. mume* bud dormancy through changes of ABA content.

## Data Availability Statement

The raw data supporting the conclusions of this article will be made available by the authors, without undue reservation.

## Author Contributions

PL, TZ, and QZ conceived and designed the experiments. PL and ZZ performed the experiments. PL, WL, ZZ, JW, TC, and LQ analyzed the data. PL and TZ wrote the manuscript. All the authors approved the final manuscript.

## Conflict of Interest

The authors declare that the research was conducted in the absence of any commercial or financial relationships that could be construed as a potential conflict of interest.

## Footnotes

1 https://www.omicshare.com/tools/Home/Soft/gogseasenior

2 https://www.omicshare.com/tools/Home/Soft/pathwaygseasenior

3 https://sg.idtdna.com/scitools/Applications/RealTimePCR/

## Supplementary Material

Supplementary Figure 1 Analysis of the scale-free fit index (left) and the mean connectivity (right) for various soft-thresholding powers.

Supplementary Figure 2 The interactive relationship network of 400 genes.

Supplementary Table 1 Primer sequences used for qRT-PCR.

Supplementary Table 2 Cellular component Gene Ontology terms for genes in the yellow module.

Supplementary Table 3 Molecular function Gene Ontology terms for genes in the yellow module.

Supplementary Table 4 Biological process Gene Ontology terms for genes in the yellow module.

Supplementary Table 5 Cellular component Gene Ontology terms for genes in the turquoise module.

Supplementary Table 6 Molecular function Gene Ontology terms for genes in the turquoise module.

Supplementary Table 7 Biological process Gene Ontology terms for genes in the turquoise module.

Supplementary Table 8 Kyoto Encyclopedia of Genes and Genomes analysis for genes in the yellow module.

Supplementary Table 9 Kyoto Encyclopedia of Genes and Genomes analysis for genes in the turquoise module.
