# Supplementary material for: Integrative Identification of Crucial Genes Associated With Plant Hormone-Mediated Bud Dormancy in Prunus mume
Source: Front Genet. 2021 Jul 6;12:698598. doi: 10.3389/fgene.2021.698598 (PMC8290171; doi:10.3389/fgene.2021.698598)
Supplement: Supplementary Figure 1 — Analysis of the scale-free fit index (left) and the mean connectivity (right) for various soft-thresholding powers. [file Data_Sheet_1.docx]

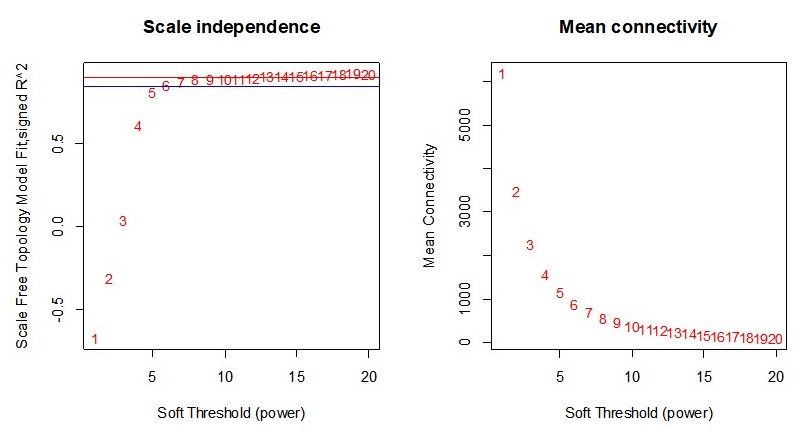


Fig. S1 Analysis of the scale-free fit index (left) and the mean connectivity (right) for various soft-thresholding powers


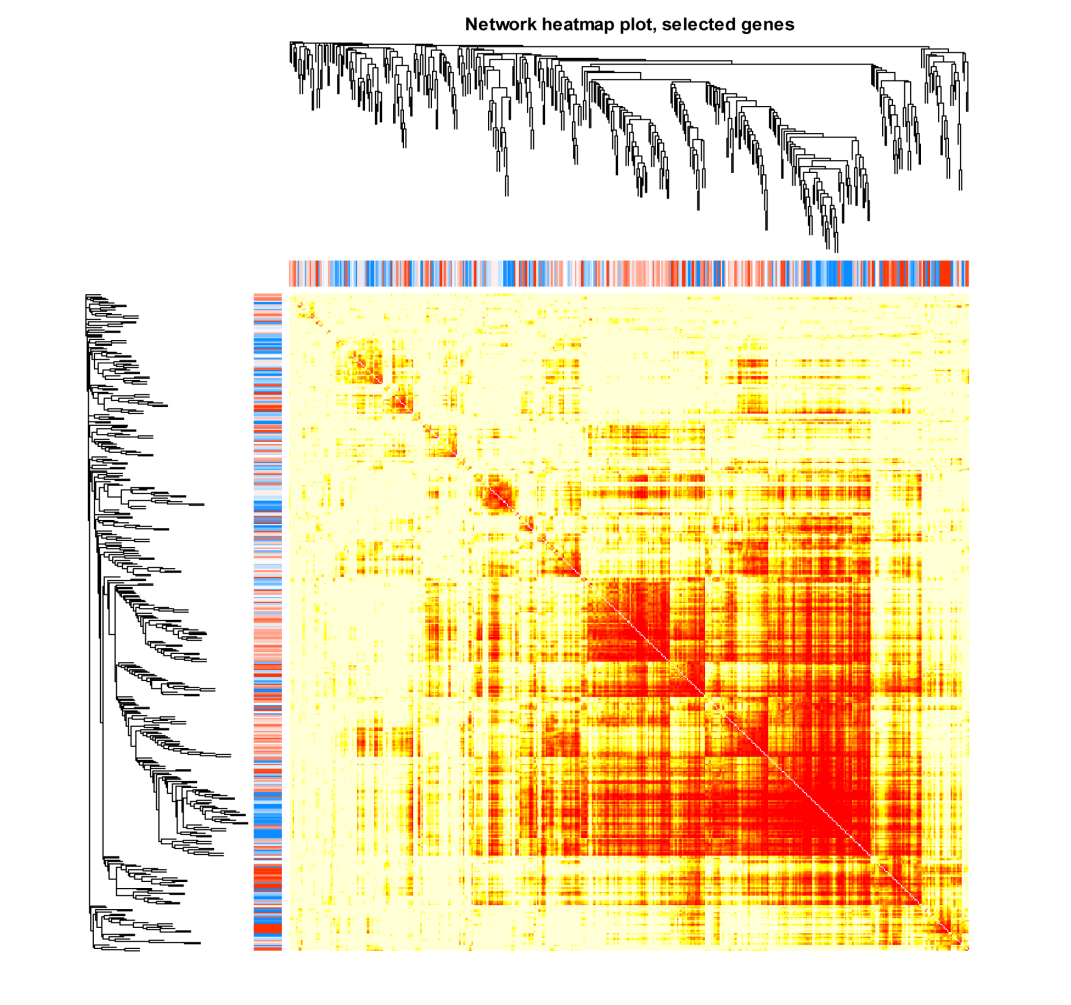


Fig. S2 The interactive relationship network of 400 genes
